# Rock Powder Solubilization and Growth Promotion of *Passiflora edulis* Seedlings by *Bradyrhizobium viridifuturi*

**DOI:** 10.1007/s00284-026-04848-8

**Published:** 2026-04-04

**Authors:** Caliane da Silva Braulio, Flávia Melo Moreira, Fernando Haddad, Silvia Maria Oliveira Longatti, Andreza Jesus de Correia, Fatima Maria de SouzaMoreira, Rafaela Simão Abrahão Nóbrega

**Affiliations:** 1 Center for Agrarian, Environmental, and Biological Sciences (CCAAB), Federal University of Recôncavo da Bahia, Cruz das Almas, Bahia Brazil; 2 Brazilian Agricultural Research Corporation (Embrapa) Cassava and Fruits, Cruz das Almas, Bahia Brazil; 3 Department of Soil Science, Federal University of Lavras, Lavras, Minas Gerais Brazil

## Abstract

The utilization of plant growth-promoting microorganisms (PGPMs) represents a promising and environmentally sustainable approach to enhance global food production. These microorganisms improve nutrient availability by solubilizing minerals such as phosphates and silicates, especially when used in combination with organic fertilizers, thereby stimulating plant development. This study aimed to evaluate the rock powder solubilization capacity of PGPMs in vitro and their agronomic efficiency when combined with organic fertilizers on the growth of *Passiflora edulis* seedlings. Four bacterial strains *Bradyrhizobium viridifuturi*,* Achromobacter spanius* and *Bacillus cereus*. were characterized based on genetic, biochemical, and physiological traits, including hydrolytic enzyme production and their ability to solubilize rock powder. Parameters influencing solubilization, such as pH and electrical conductivity, were also evaluated. Greenhouse experiments were conducted over 60 and 90 days to assess seedling growth under four substrate conditions, with and without chemical fertilization. Growth parameters, including plant height, stem diameter, leaf number, and nutrient accumulation, were measured. *(A) spanius* and *(B) cereus*, effectively solubilized silicate rock in vitro. Notably, *B. viridifuturi* significantly enhanced seedling growth and nutrient uptake when applied to substrates containing both organic fertilizer and rock powder. These findings underscore the potential of PGPMs particularly *B. viridifuturi* as sustainable bioinoculants to improve nutrient availability and optimize seedling development in agricultural systems.

## Introduction

Yellow passion fruit (*Passiflora edulis* Sims) plays a key role in Brazil’s agricultural economy, being one of the principal tropical fruits cultivated for both domestic consumption and export. In 2021, national production reached 697,859 tons [24]. However, maintaining high productivity and fruit quality remains a challenge, particularly due to the dependence on seedling vigor and appropriate fertilization practices [46, 53].

To reduce reliance on synthetic fertilizers without compromising crop performance, the use of plant growth-promoting bacteria (PGPB) has emerged as a promising and sustainable alternative [51]. These beneficial bacteria enhance plant growth by increasing nutrient availability, inducing plant defense mechanisms, and promoting tolerance to biotic and abiotic stresses. Their effects are commonly observed in improvements in plant height, stem thickness, leaf number, biomass accumulation, root development, and nitrogen uptake [8, 37, 38, 41, 46, 52].

PGPBs exert these effects through mechanisms such as the production of phytohormones (e.g., auxins), organic acids, siderophores, and extracellular enzymes (e.g., urease, phosphatases, proteases), which contribute to nutrient solubilization and uptake [15, 29, 38, 41, 51].

The use of remineralizing rock powders in agriculture has gained prominence as a sustainable practice for improving soil fertility and enhancing plant nutrition. These materials, derived from ground rocks, are natural sources of essential nutrients such as phosphorus (P), potassium (K), and silicon (Si), which play critical roles in plant growth, development, and responses to adverse environmental conditions. Silicon, in particular, has been widely recognized for its ability to increase plant resistance to both biotic and abiotic stresses. Through bacterial weathering of silicate minerals, soluble silicon is released into the soil, improving nutrient availability and overall plant performance [17]. The ability of silicate-solubilizing bactéria, notably species belonging to the genera *Bacillus*,* Burkholderia*, *Rhizobium*, and *Pseudomonas* to mobilize Si and K from rock powders represents a sustainable alternative that can reduce dependency on synthetic fertilizers [17, 60].

However, the efficiency of SSB inoculation depends on interactions between microbial traits, plant genotype, and substrate nutrient content. Therefore, this study aimed to assess the in vitro rock powder solubilization potential of selected PGPBs and their agronomic efficiency, in combination with organic fertilizers, on the growth and nutrition of *P. edulis* seedlings.

## Materials and Methods

### Identification and Characterization of Bacterial Strains

Bacterial strains from the microorganism collections of the Federal University of Recôncavo da Bahia (UFRB), Cruz das Almas, BA, and the Federal University of Lavras (UFLA), Lavras, MG, Brazil, were reactivated on synthetic nutrient agar (SNA) and incubated at 28 ± 1 °C under a 12-hour photoperiod. Subsequently, the strains were cultured in nutrient broth for the extraction of genomic DNA encoding the 16 S rRNA gene. DNA extraction was performed following the protocol described by Sambrook et al. [48]. Amplification was conducted using a GeneAmp PCR System 9700 (Applied Biosystems), and the purified PCR amplicons were sequenced at Eurofins Genomics India (Bengaluru). The resulting sequences were analyzed using BLAST against reference sequences available in the NCBI GenBank database [4]. The bacterial strains *Bradyrhizobium viridifuturi* – SEMIA 6461 [16], UFRB FA72A2-1 and UFRB FA34C2-2 were selected based on previous studies, as presented in Table 1.

**Table 1:** Origin, characteristics, hosts, and functions of the bacterial strains

Strains	Origin		Characteristics	Hosts	Functions
*Bradyrhizobium viridifuturi* – UFLA 03–84 (SEMIA 6461)		Isolated from soils of the Brazilian Amazon; obtained from the collection of the Soil Science Department at the Federal University of Lavras, authorized by the Ministry of Agriculture, Livestock, and Food Supply (MAPA) [11] as an inoculant for cowpea.	Slow growth of 4 to 6 days.	Cowpea [*Vigna unguiculata* (L.) Walp.] [15, 27, 54].	Biological nitrogen fixation, plant growth promotion, nitrogen accumulation in the aerial parts, and solubilization of CaHPO₄ and Al(H₂PO₄)₃ [10, 34, 35, 56].
UFRB FA34C2-2		Isolated from an area with a history of cassava effluent application in the municipality of Vitória da Conquista, Brazil [55]	Rapid growth − 1 to 2 days.	Cowpea [55].	Plant growth promotion, biological nitrogen fixation [9, 10, 14, 41].
UFRB FA72A2-1		Isolated from an area with a history of cassava effluent application in the municipality of Vitória da Conquista [55].	Rapid growth – 1 to 2 days.	Cowpea [55].	Solubilization of CaHPO_4_ [55]; Plant growth promotion [41]

After reactivation, the bacterial strains were evaluated for their ability to synthesize auxins, siderophores, and extracellular hydrolytic enzymes, including urease, catalase, cellulase, protease, pectinase, and amylase [3, 12, 22, 28, 30, 40]. Biochemical and physiological analyses were performed in triplicate at the Soil Microbiology Laboratory of Embrapa Cassava and Tropical Fruits

### In Vitro Solubilization of Rock Powder

To assess the ability of bacterial strains to solubilize rock powder, an in vitro experiment was conducted using a completely randomized design arranged in a 4 × 2 factorial scheme with three replicates in 150 mL Erlenmeyer flasks. The factors consisted of: (1) bacterial treatments—control (uninoculated), UFRB FA34C2-2, UFRB FA72A2-1 and UFLA 03–84, and (2) rock powder (SRP) doses—absence (0 g) and presence (0.5 g). Evaluations were conducted on days 7, 21, and 35.

The silicate rock powder (Ipirá Fértil) used in the experiment is derived from pyroxenite rock and was sourced from the region of Ipirá, Bahia, Brazil. The physicochemical analysis was provided by SGS GEOSOL LABORATÓRIOS LTDA (Report CA n° GQ2104274, REV.:00, issued on 16 July 2021). According to the laboratory report, the rock powder had the following chemical composition (%): SiO₂: 58.3; Al₂O₃: 13.3; Fe₂O₃: 7.15; CaO: 6.09; MgO: 4.36; TiO₂: 0.47; P₂O₅: 0.73; Na₂O: 1.36; K₂O: 5.44; MnO: 0.24; and Loss on Ignition (LOI): 3.04. The particle size was 0.3 mm, according to the manufacturer.

The bacterial strains were cultured in 50 mL of nutrient broth containing 5 g NaCl, 5 g peptone, 1.5 g meat extract, and 1.5 g yeast extract per 1000 mL of distilled water (Merck, Germany). The nutrient broth without inoculum or rock powder presented initial concentrations of Na⁺ (5.03 mg L⁻¹), Si (1.12 mg L⁻¹), and K⁺ (0.21 mg L⁻¹). The initial pH and electrical conductivity (σ) of the medium with and without rock powder were 6.8 and 8.2 µS cm⁻¹, and 6.0 µS cm⁻¹ and 8.0 µS cm⁻¹, respectively.

For inoculated treatments, 1 mL of bacterial suspension (1 × 10⁸ CFU mL⁻¹) was added to the culture medium. The nine flasks para cada tratamento were incubated at 30 °C with constant shaking at 160 rpm for 35 days. Measurements of pH and electrical conductivity were taken em três flasks on days 7, em três flasks 21, and três flasks 35. On the final day, the culture media were filtered using Whatman No. 01 filter paper under vacuum, and the concentrations of K⁺ and Na⁺ in the filtrate were determined by flame photometry, while Si was measured using atomic absorption spectrophotometry. Element concentrations were expressed in mg L⁻¹.

### Effect of Bacterial Inoculation on Seedling Growth and Nutrition

To evaluate the effects of bacterial inoculation on the growth and nutrient status of *Passiflora edulis* seedlings, two greenhouse experiments were conducted at the experimental farm of the Center for Agricultural, Environmental, and Biological Sciences (CCAAB), Federal University of Recôncavo da Bahia (UFRB), in Cruz das Almas, Bahia, Brazil (12°39’27” S, 39°04’58” W; 215 m altitude).

In the first experiment, seeds were sown in polyethylene tubes with a capacity of 280 cm³ and cultivated for 60 days after sowing (DAS). In the second experiment, the seeds were sown in polyethylene bags with a capacity of 1.02 dm³, and the cultivation period was extended to 90 DAS.

### Preparation of Inoculum and Growing Substrates

For inoculum preparation, the strain UFLA 03–84 was cultivated in semi-solid 79 medium for four days [36], while the strains UFRB FA34C2-2 and UFRB FA72A2-1, were grown in liquid nutrient broth for two days at 25 °C, reaching the logarithmic growth phase (1 × 10⁸ CFU mL⁻¹).

The growing substrates consisted of the following combinations: (1) soil + organic fertilizer (2:1, v/v) + rock powder; (2) soil + organic fertilizer (2:1, v/v); (3) soil + rock powder; and (4) soil alone. The soil used in both experiments—evaluated at 60 and 90 days after sowing (DAS) was a Dystrophic Yellow Latosol collected from the subsurface layer (> 40 cm) on the UFRB campus. Its chemical and physical properties were as follows: pH (H₂O): 5.0; pH (KCl): 4.0; ΔpH: 1.1; organic matter (OM): 1.43 kg kg⁻¹; available P (Mehlich 1): 0.04 mg dm⁻³; K⁺: 39.01 mg dm⁻³; Ca²⁺: 0.7 cmol_c_ dm⁻³; Mg²⁺: 0.6 cmol_c_ dm⁻³; (H + Al): 1.9 cmol_c_ dm⁻³; Na: 0.17 cmol_c_ kg⁻¹; sum of bases (SB): 40.32 cmol_c_ dm⁻³; effective CEC: 1.81 cmol_c_ dm⁻³; potential CEC: 3.21 cmolc dm⁻³; base saturation (V): 40.81%; sand: 514 g kg⁻¹; silt: 104 g kg⁻¹; clay: 382 g kg⁻¹; clay dispersion: 94 g kg⁻¹; flocculation degree: 193 g kg⁻¹. Soil electrical conductivity was 0.13 dS m⁻¹.

The organic fertilizer used in the substrates was produced at UFRB from composted tree prunings and manure from cattle and goats (3:1:1 ratio). Its composition was: pH (CaCl₂ 0.01 M): 7.6; total OM: 13.96%; N: 0.60%; P₂O₅: 1.36%; K₂O: 0.75%; Ca: 0.98%; Mg: 0.21%; S: 0.07%; C/N ratio: 9.0; Cu: 2 mg kg⁻¹; Mn: 200 mg kg⁻¹; Zn: 85 mg kg⁻¹; Fe: 10,089 mg kg⁻¹; B: 5 mg kg⁻¹; Na: 1,274 mg kg⁻¹. The composition of the silicate rock powder was previously described (see Sect. 2.1). Chemical fertilization in the control treatment (soil only) was applied based on the crop’s nutritional requirements during the sowing stage: 150 kg ha⁻¹ of N, 120 kg ha⁻¹ of P, and 20 kg ha⁻¹ of K [7].

### Experimental Design

In the first experiment, a completely randomized design was used in a 5 × 4 factorial arrangement (inoculation sources × substrates), with 10 replicates and one plant per polyethylene tube. The treatments included: Factor 1- no inoculation and no chemical fertilization- No Che (SI) (i), no inoculation with chemical fertilization - Whit Che (SF) (ii), and inoculation with one of three bacterial strains UFLA 03–84 (iii), UFRB FA34C2-2 (iv), or UFRB FA72A2-1 (v); and Factor 2- four substrate compositions: soil + organic fertilizer + rock powder (i); soil + organic fertilizer (ii); soil + rock powder (iii); and soil alone (iv).

To validate the results obtained in the first experiment, a second experiment was conducted using a similar design, arranged in a 5 × 4 factorial scheme (five inocula × four substrates), with eight replicates and one plant per 1.02 dm³ polyethylene bag.

### Planting, Inoculation, and Fertilization

Two seeds of *Passiflora edulis* Sims (accession BGP190 from the Embrapa Cassava and Fruits Germplasm Bank) were sown per container at a depth of 1 cm. Inoculation was carried out by applying 1 × 10⁸ CFU mL⁻¹ of bacterial suspension directly to the disinfected seeds. Control treatments received only the sterile culture medium (nutrient broth composed of 5 g NaCl, 5 g peptone, 1.5 g meat extract, and 1.5 g yeast extract per 1000 mL of distilled water; Merck, Germany), without inoculation.

In the chemical fertilization treatment, 41 mg dm⁻³ of N (urea), 18 mg dm⁻³ of P₂O₅ (triple superphosphate), and 4.8 mg dm⁻³ of K₂O (potassium chloride) were applied in the first experiment. In the second experiment, doses were increased to 48 mg dm⁻³ of N, 41 mg dm⁻³ of P₂O₅, and 21 mg dm⁻³ of K₂O. In treatments without chemical fertilization, P₂O₅ and K₂O were omitted to evaluate the isolated effects of inoculation, organic fertilizer, and/or silicate rock powder.

For treatments with rock powder, 0.48 g dm⁻³ (tubes) or 2.08 g dm⁻³ (bags) of the material was incorporated into the substrate [31]. The fertilizer doses in both experiments were adjusted based on container volume. All plants were irrigated daily under greenhouse conditions. Thinning was performed 15 days after sowing.

### Morphological and Nutritional Characteristics of Passion Fruit Seedlings

In Experiments 1 and 2, seedlings were evaluated at 60 and 90 days after sowing (DAS), respectively, for the following parameters: plant height, stem diameter, number of leaves, root length, and chlorophyll content (chlorophyll a, chlorophyll b, and total chlorophyll a + b). Chlorophyll content was measured using a portable chlorophyll meter (ClorofiLOG CFL 1030).

Following these measurements, shoots and roots were separated and dried in a forced-air oven at 65 °C for 48 h. The dry mass of shoots, roots, and total biomass was determined, and the Dickson Quality Index (DQI) was calculated as proposed by Dickson et al. [19].

Dried shoot samples from three seedlings per treatment were selected for nutrient analysis. Nitrogen (N), phosphorus (P), and potassium (K) concentrations (%) were determined following the protocols described by Tedesco, Volkweiss, and Bohnen [57]. The accumulations of N, P, and K (mg per plant) were calculated by multiplying the shoot dry mass by the corresponding nutrient concentration.

### Reisolation of Bacterial Strains

To validate the causal relationship, the bacterial strains were reisolated from inoculated plants following the observation of beneficial effects on growth and nutritional status. No strains were recovered from non-inoculated controls. These findings confirm that the observed improvements can be attributed to the inoculation with the tested strains.

### Statistical Analysis

In the in vitro solubilization experiment, means corresponding to rock powder doses were compared using the F-test at a 5% significance level. All data were subjected to analysis of variance (ANOVA), and treatment means were grouped using the Scott–Knott test at the 5% probability level. For the in vivo experiments (Experiments 1 and 2), treatment means were compared using the Tukey test at a 5% significance level. All statistical analyses were performed using the R statistical software [47].

## Results

### Identification and Characterization of Bacterial Strains

All bacterial strains were capable of producing auxin and the enzyme amylase. Strain UFRB FA34C2-2 exhibited outstanding activity in producing lytic enzymes, including catalase, amylase, and protease. Strain UFRB FA72A2-1 produced cellulase, amylase, and protease. None of the strains were able to produce siderophores or the enzyme pectinase (Table 2).

**Table 2:** Biochemical and physiological of the bacterial strain

Characteristics	A. spanius	B. cereus	B. viridifuturi
	UFRB FA34C2-2	UFRB FA72A2-1	UFLA-0384
Siderophore	**-**	**-**	**-**
Auxin	**+**	**+**	**+**
Urease	**-**	**-**	**-**
Cellulase	**-**	**+**	**-**
Catalase	**+**	**-**	**+**
Amylase	**+**	**+**	**+**
Pectinase	**-**	**-**	**-**
Protease	**+**	**+**	nd

+ (produces); - (not produces); nd (not determined)

Sequencing of the DNA region encoding the 16 S rRNA gene indicated that strain UFRB FA34C2-2 (PQ219077.1) belongs to the genus *Achromobacter*, showing 99.3% similarity to *Achromobacter spanius* LMG 5911^T (AY_170848.1), its closest type strain. Similarly, strain UFRB FA72A2-1 (PQ219078.1) was identified as a member of the genus *Bacillus*, with 99.5% similarity to *Bacillus cereus* ATCC 14,579^T (MH_806388.1). The sequences obtained were analyzed using the BLAST tool against reference sequences available in the NCBI GenBank database. The identification of both strains was based on sequence similarity to their respective type strains and further supported by the comparison of additional housekeeping genes, conducted using PCR-amplified sequences.

### In Vitro Solubilization of Rock Powder by Bacterial Strain*s*

**Fig. 1:**
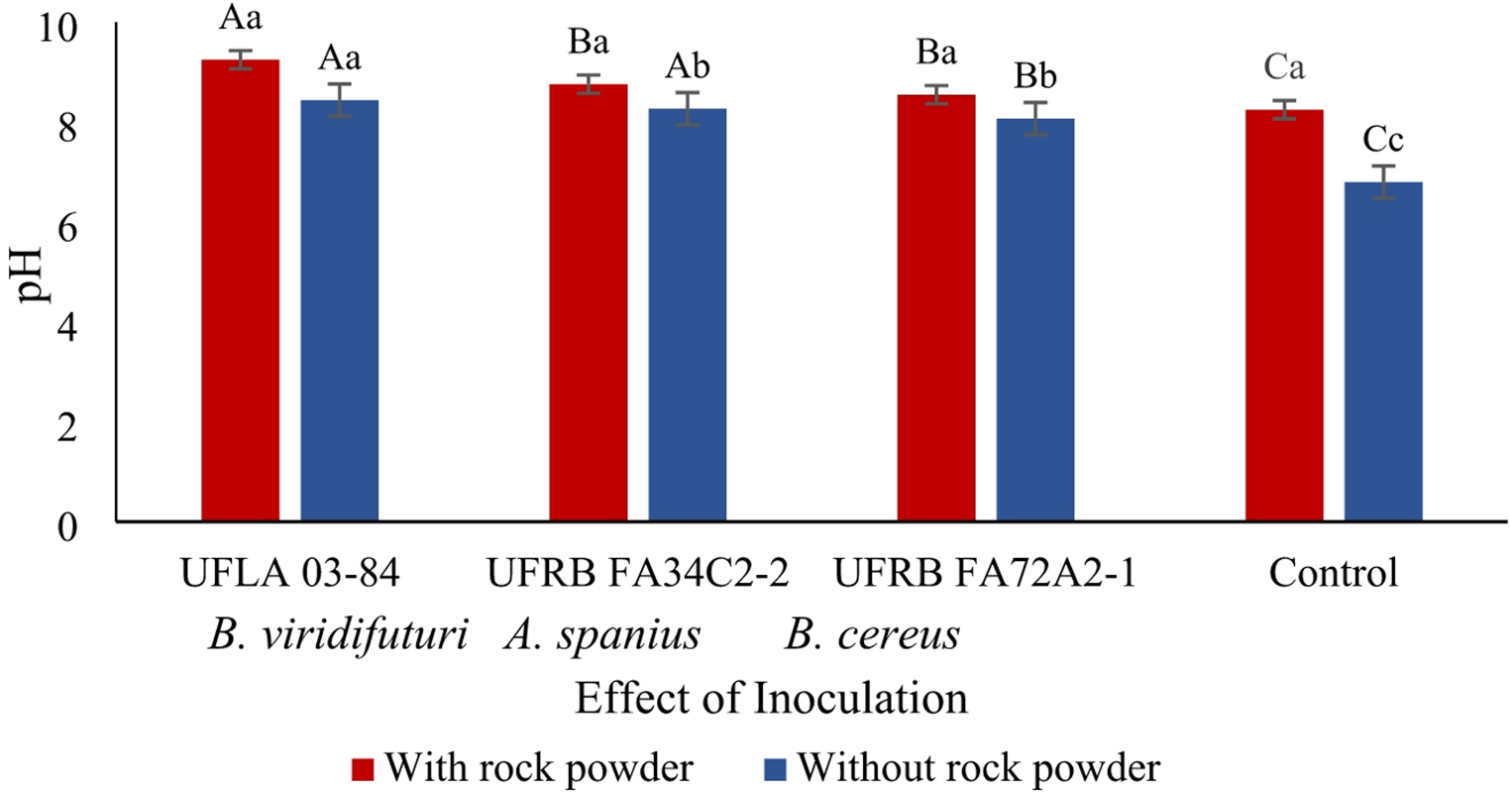
pH of the culture medium under the effect of bacterial inoculation in the presence and absence of rock powder. Means followed by the same uppercase letter (for bacterial strains) or lowercase letter (for rock powder treatments) did not differ significantly according to the Scott–Knott or F-test at the 5% probability level

A significant three-way interaction was observed among bacterial inoculation, the presence or absence of rock powder, and the evaluation period for electrical conductivity. Additionally, a two-way interaction between the inoculants and the presence or absence of rock powder significantly affected pH, sodium, and silicon concentrations. The pH was influenced by interactions between the bacterial strains and rock powder, as well as between rock powder and the evaluation periods (*p* < 0.05) (Fig. 1).

Treatments inoculated with *B. viridifuturi* UFLA 03–84, both in the presence and absence of rock powder, exhibited the highest average pH values. In contrast, the control treatment (without rock powder) showed the lowest pH values compared to the other treatments (Fig. 1).

**Fig. 2:**
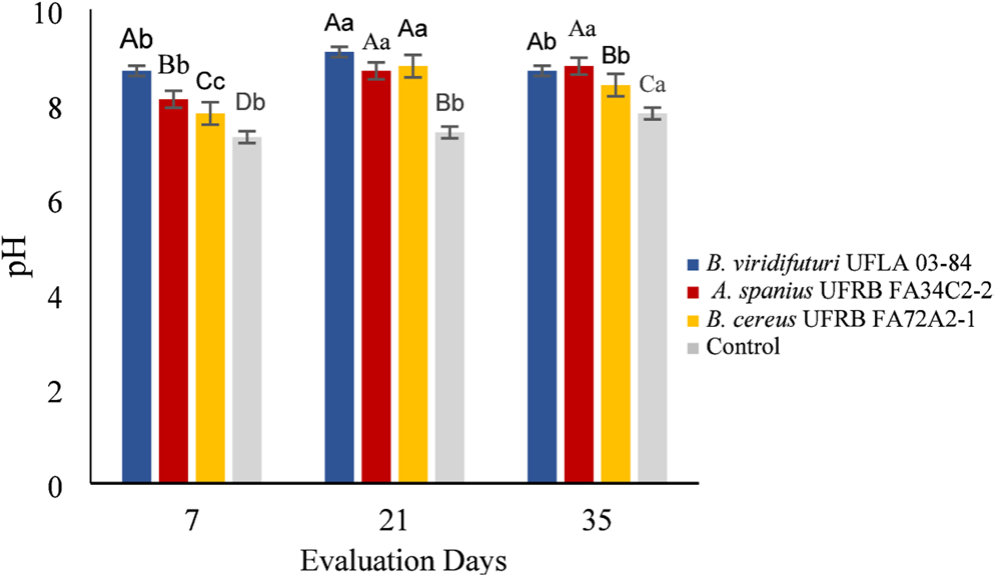
pH of the culture medium under the effect of inoculation with bacterial strains at different fermentation times. Means followed by the same uppercase letter (for bacterial strains) or lowercase letter (for evaluation days) did not differ significantly according to the Scott–Knott or F-test at the 5% probability level

On the 7th day of fermentation, the highest pH was observed in the culture medium inoculated with *B. viridifuturi* UFLA 03–84. Media inoculated with *B. cereus* presented the lowest pH values (7.8), while the control maintained the initial pH level (Fig. 2). From the 7th to the 21 st day, the pH increased across all treatments, with *B. viridifuturi* UFLA 03–84 reaching the highest value. By the 21 st day, strains *(A) spanius* UFRB FA34C2-2 and *(B) cereus* UFRB FA72A2-1, exhibited similar pH values, while the control recorded the lowest (6.8). On the 35th day, pH levels decreased in all treatments, except for *(A) spanius* UFRB FA34C2-2 and *(B) viridifuturi* UFLA 03–84, which maintained the highest averages. Interestingly, the control treatment showed an increase in pH, reaching 6.8.

**Fig. 3:**
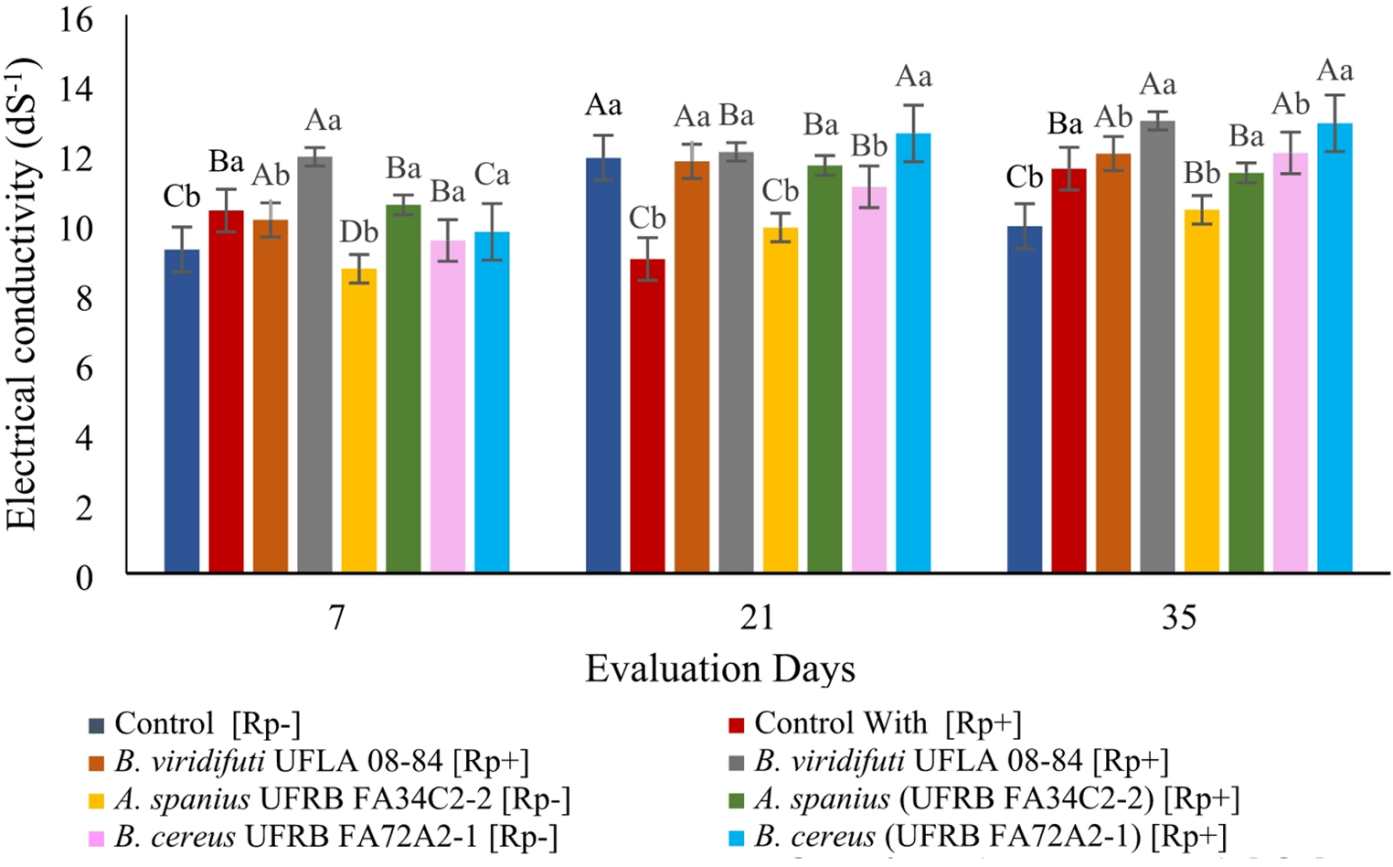
Electrical conductivity of the culture medium under the effects of bacterial inoculation and rock powder at different fermentation times. Means followed by the same uppercase letter (for bacterial strains) or lowercase letter (for evaluation times) did not differ significantly according to the Scott–Knott or F-test at the 5% probability level

Electrical conductivity was influenced by the treatments and their interactions. A continuous increase in conductivity was observed in the treatments inoculated with *B. viridifuturi* UFLA 03–84, *(A) spanius* UFRB FA34C2-2 and *(B) cereus* UFRBFA72A2-1, both in the presence and absence of rock powder. In the control treatment with rock powder, conductivity decreased on the 21 st day and increased again on the 35th day. In the absence of rock powder, conductivity peaked on the 21 st day and remained unchanged between the 7th and 35th days (Fig. 3).

**Fig. 4:**
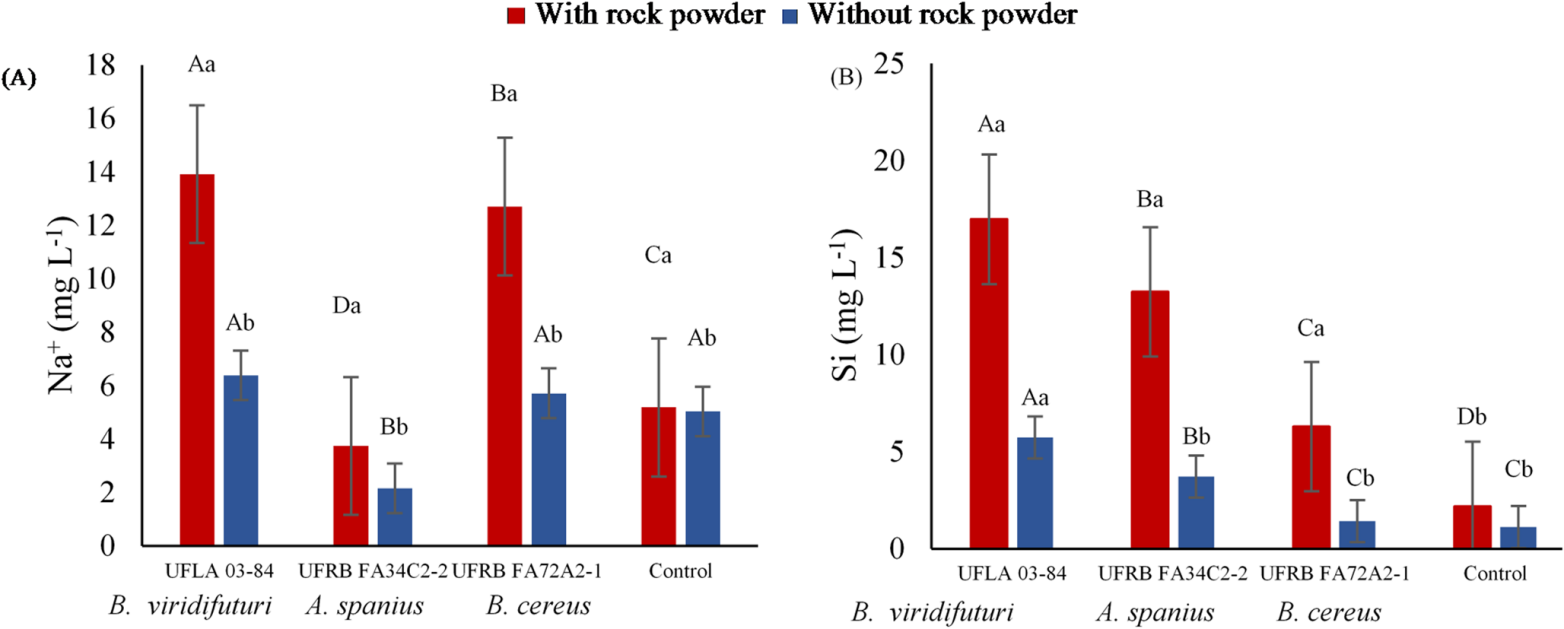
(**A**) Concentration of sodium (Na⁺) and (**B**) silicon (Si) available in the liquid culture medium with and without rock powder under the influence of bacterial inoculation on the 35th day. Means followed by the same uppercase letter (for bacterial strains) or lowercase letter (for rock powder treatments) did not differ significantly according to the Scott–Knott or F-test at the 5% probability level

The highest average concentrations of Na⁺ and Si were recorded in treatments with silicate rock powder, particularly in those inoculated with *B. viridifuturi* UFLA 03–84, both in the presence and absence of rock powder. This was followed by *B. cereus* UFRB FA72A2-1 for Na⁺ concentration and *A*. *spanius* UFRB FA34C2-2 for Si concentration (Fig. 4).

**Fig. 5:**
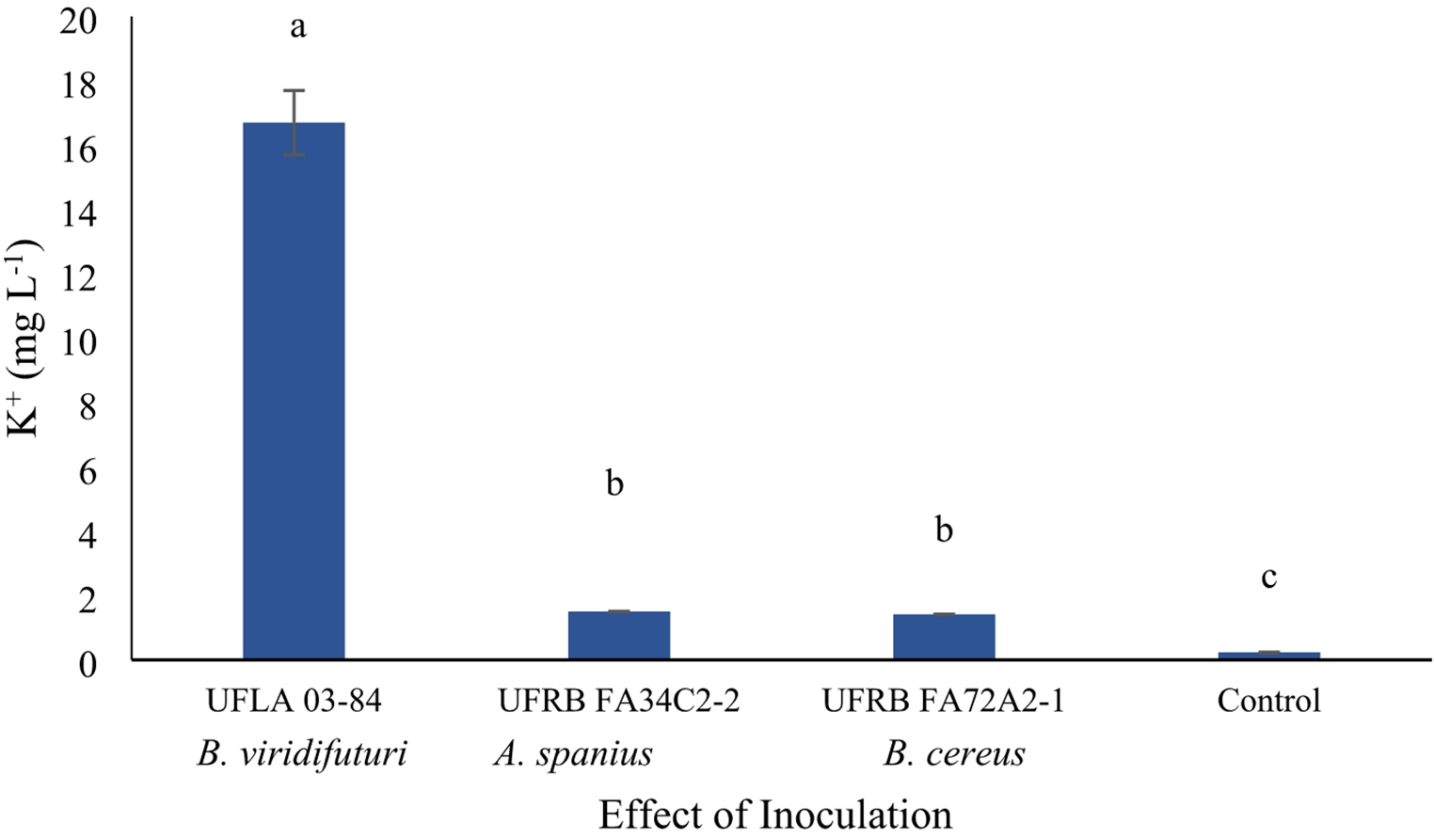
Concentration of available potassium (K⁺) in the liquid culture medium under the influence of bacterial inoculation at 35 days. Means were grouped using the Scott–Knott test at a 5% probability level

A significant interaction (*p* < 0.05) was observed between the bacterial inocula and the presence of rock powder, influencing the concentration of K⁺. However, in the absence of rock powder, no significant effect (*p* > 0.05) on K⁺ concentration was detected, except for the treatment with *B. viridifuturi* UFLA 03–84, which exhibited the highest K⁺ concentration (Fig. 5).

### Inoculation Experiments with Bacterial Strains in Seedlings Grown in Substrates

In both the first and second experiments, significant interaction (*p* < 0.05) was observed between cultivation substrates and bacterial inocula for all biometric variables (Tables 3 and 4). Seedlings inoculated with *B. viridifuturi* UFLA 03–84, followed by *A.* spanius UFRB FA34C2-2, exhibited the highest averages for plant height, number of leaves, and root length in both experiments. The most significant results were observed when seedlings were grown in soil + organic fertilizer + silicate rock powder and in soil + organic fertilizer, respectively, at 60 and 90 days after sowing (DAS).

Seedlings inoculated with *(A) spanius* UFRB FA34C2-2 also showed significantly higher values for these variables when compared to those inoculated with *(B) cereus* UFRB FA72A2-1 (Tables 3 and 4).

Seedlings that received no bacterial inoculation or fertilization, as well as those cultivated in soil + rock powder and in pure soil, presented reduced values for number of leaves and root length, regardless of the cultivation substrate used (Table 3).

**Table 3:** Averages of the biometric variables of *Passiflora edulis* Sims seedlings based on the interaction between the cultivation substrate and inoculum, at 60 days after sowing on polyethylene tubes

Subtrates		Inoculum			
	B. viridifuturi	B. cereus	A. spanius	Whit Che	No Che
Height (cm plant^− 1^)					
SFR	20.16Aa	17.88Ca	18.59Ba	17.80Cc	17.02Da
SF	18.12Bb	16.47Bb	16.98Bb	16.89Bb	15.70Cb
SR	14.89Ac	11.85Bc	11.68Bc	11.90Bc	8.84Cc
Pure soil	14.02Ad	8.77Bd	9.01Bd	9.19Bd	7.48Cd
CV (%)	3.82	3.82	3.82	3.82	3.82
Leaf number (leaves plant⁻¹)					
SFR	8.1Aa	7.0Aa	6.7Ba	6.5Ba	6.8Ba
SF	7.0Ab	6.5Aa	6.6Aa	6.5Aa	6.8Aa
SR	6.6Ab	5.3Bb	5.1Bb	5.2Bb	4.8Bb
Pure soil	6.1Ac	4.8Bb	5.1Bb	5.3Bb	4.5Bb
CV (%)	11.31	11.31	11.31	11.31	11.31
Root length (cm plant⁻¹)					
SFR	21.15Aa	19.53Ba	19.55Ba	17.81Ca	16.69Da
SF	20.09Ab	19.00Ba	19.88Aa	17.00Ca	14.81Db
SR	20.32Ab	17.87Bb	20.32Aa	15.89Cb	13.06Dc
Pure soil	16.98Bc	15.58Cc	17.95Ab	15.52Cb	12.26Dc
CV (%)	5.3	5.3	5.3	5.3	5.3
Shoot dry mass (g plant⁻¹)					
SFR	0.99Aa	0.50Da	0.60Ca	0.91Ba	0.50Da
SF	0.89Ab	0.50Db	0.50Cb	0.51Bb	0.40Cb
SR	0.59Ac	0.26Cc	0.20Dc	0.30Bc	0.18Ec
Pure soil	0.40Ad	0.16Cd	0.16Dd	0.20Bd	0.13Ed
CV (%)	0.86	0.86	0.86	0.86	0.86
Root dry mass (g plant⁻¹)					
SFR	0.60Aa	0.40Ca	0.40Ca	0.50Ba	0.29Db
SF	0.50Ab	0.39Cb	0.40Ba	0.40Bb	0.25Da
SR	0.39Ac	0.14Cc	0.09Db	0.20Bc	0.08Ec
Pure soil	0.28Ad	0.08Cd	0.07Dc	0.10Bd	0.06Ed
CV (%)	0.96	0.96	0.96	0.96	0.96
Total dry mass (g plant⁻¹)					
SFR	1.59Aa	0.90Da	1.00Ca	1.41Ba	0.79Ea
SF	1.39Ab	0.88Db	0.90Cb	0.91Bb	0.66Fb
SR	0.97Ac	0.39Cc	0.29Dc	0.50Bc	0.26Ec
Pure soil	0.68Ad	0.24Cd	0.22Dd	0.30Bd	0.19Ed
CV (%)	0.62	0.62	0.62	0.62	0.62
Dickson Quality Index					
SFP	0.31Aa	0.17Ca	0.18Ca	0.25Ba	0.12Da
SF	0.26Ab	0.14Cb	0.14Cb	0.17Bb	0.10Da
SR	0.17Ac	0.07Cc	0.06Cc	0.11Bc	0.07Cb
Pure soil	0.06Ad	0.07Ac	0.05Bc	0.07Ad	0.04Bc
CV (%)	15.78	15.78	15.78	15.78	15.78

SFR = soil + organic fertilizer + rock powder; SF = soil + organic fertilizer; SR = soil + rock powder. Means followed by the same uppercase letter in a row belong to the same group according to the Scott–Knott test at the 5% probability level; means followed by the same lowercase letter in a column do not differ significantly according to the Tukey test at the 5% probability level. With Che: with chemical fertilization; No Che: without chemical fertilization and without inoculation

For shoot dry mass, root dry mass, total dry mass, and the Dickson Quality Index (DQI), seedlings inoculated with *B. viridifuturi* UFLA 03–84 and cultivated in substrates composed of soil + organic fertilizer + rock powder and soil + organic fertilizer exhibited significantly higher averages than the other treatments, followed by the treatment with chemical fertilization (*p* < 0.05), at 60 and 90 days after sowing (DAS). Seedlings grown in soil + organic fertilizer + rock powder and inoculated with *(A) spanius* UFRB FA34C2-2 and *(B) cereus* UFRB FA72A2-1 also exhibited significantly higher values for these variables when compared to the uninoculated control and the treatment with soil alone (Tables 3 and 4).

In Experiment 2, the highest averages were observed in plants cultivated in the substrate composed of soil + organic fertilizer + rock powder and inoculated with *B. viridifuturi* UFLA 03–84 and *A. spanius* UFRB FA34C2-2, as well as in the treatment with chemical fertilization; no significant differences were found among these three treatments (Table 4).

**Table 4:** Averages of the growth variables of *Passiflora edulis* Sims based on the cultivation substrate and inoculum, at 90 days after sowing on polyethylene bags

Substrates			Inoculum		
	B. viridifuturi UFLA 0384	A. spanius UFRB FA34C2-2	B. cereusUFRB FA72A2-1	With Che	No Che
Height (cm plant-1)					
SFR	29.91Aa	29.03Ba	26.18Da	28.54Da	17.51Ea
SF	28.96Ab	27.03Bb	24.96Cb	24.64Cb	16.95Db
SR	23.01Ac	22.23Bc	20.29Dc	14.05Dc	9.35Ec
Pure soil	21.80Ad	21.58Ad	15.01Bd	11.80Cd	8.74Dd
CV (%)	2.05	2.05	2.05	2.05	2.05
Leaf number (leaves plant⁻¹)					
SFR	11.25Aa	10.50Ba	9.50Ca	9.38Ca	7.38Da
SF	9.50Ab	9.50Ab	9.13Aa	8.63Ba	7.8Ca
SR	6.63Ac	5.63Bc	6.38Ab	6.38Ab	5.38Bb
Pure soil	5.25Ad	4.38Bd	4.75Bc	4.38Bc	4.63Bb
CV (%)	8.02	8.02	8.02	8.02	8.02
Root length (cm plant⁻¹)					
SFR	26.15Aa	25.90Aa	24.00Ca	25.24Ba	17.25Da
SF	25.05Ab	24.98Ab	23.96Ba	23.95Bb	16.35Cb
SR	23.89Ac	19.90Cc	18.00Db	22.00Bc	13.10Ec
Pure soil	22.43Ad	15.18Dd	15.18Cc	20.06Cd	12.09Ed
CV (%)	2.0	2.0s	2.0	2.0	2.0
Shoot dry mass (g plant⁻¹)					
SFR	1.91Aa	1.60Ba	1.72Ba	1.82Aa	0.51Ca
SF	1.91Aa	1.33Bb	1.21Cb	1.41Bb	0.41Da
SR	0.79Ab	0.60Bc	0.50Bc	0.68Bc	0.19Cb
Pure soil	0.50Ac	0.43Ac	0.29Bd	0.40Ad	0.17Bb
CV (%)	16.67	16.67	16.67	16.67	16.67
Root dry mass (g plant⁻¹)					
SFR	0.90Aa	0.90Aa	0.50Ba	0.90Aa	0.39Ca
SF	0.83Ab	0.73Cb	0.49Da	0.80Bb	0.35Eb
SR	0.69Ac	0.22Cc	0.24Cb	0.30Bc	0.19Dc
Pure soil	0.39Ad	0.12Cd	0.91Dc	0.20Bd	0.09Dd
CV (%)	3.4	3.4	3.4	3.4	3.4
Total dry mass (g plant⁻¹)					
SFR	2.81Aa	2.50Ba	2.22Ca	2.72Aa	0.91Da
SF	2.74Aa	2.05Cb	1.69Db	2.21Bb	0.76Ea
SR	1.47Ab	0.82Cc	0.73Cc	0.98Bc	0.38Db
Pure soil	0.88Ac	0.55Bd	0.38Cd	0.59Bd	0.27Cb
CV (%)	10.93	10.93	10.93	10.93	10.93
Dickson Quality Index					
SFR	0.34Aa	0.28Ba	0.29Ba	0.32Aa	0.15Ca
SF	0.29Ab	0.25Ba	0.21Cb	0.21Cb	0.12Da
SR	0.15Ac	0.10Bb	0.06Bc	0.15Ac	0.07Bb
Pure soil	0.09Ad	0.05Bc	0.04Bc	0.08Ad	0.05Bb
CV (%)	29.93	29.93	29.93	29.93	29.93

SFR = soil + organic fertilizer + rock powder; SF = soil + organic fertilizer; SR = soil + rock powder. Means followed by the same uppercase letter in a row belong to the same group according to the Scott–Knott test at the 5% probability level; means followed by the same lowercase letter in a column do not differ significantly according to the Tukey test at the 5% probability level. With Che: with chemical fertilization; No Che: without chemical fertilization and without inoculation

Lower averages of dry mass and Dickson Quality Index (DQI) were observed in seedlings that did not receive bacterial inoculation or chemical fertilization across all evaluated substrates (Tables 3 and 4).

Passion fruit seedlings inoculated with *B. viridifuturi* UFLA 03–84 and cultivated in a substrate composed of soil, and rock powder exhibited the highest values for chlorophyll a, chlorophyll b, and total chlorophyll content, followed by seedlings subjected to chemical fertilization, at 60 and 90 days after sowing (DAS) (Tables 5 and 6).

In Experiment 1, seedlings inoculated with *A*. *spanius* UFRB FA34C2-2 and *B. cereus* UFRB FA72A2-1 and cultivated in soil + organic fertilizer + rock powder also showed significantly higher chlorophyll levels compared to control (Table 5).

Seedlings grown in the substrate composed only of soil, without bacterial inoculation or chemical fertilization, exhibited the lowest chlorophyll values in both experiments (Tables 5 and 6).

**Table 5:** Averages of the nutritional variables of *Passiflora edulis* Sims seedlings based on the interaction between the cultivation substrate and inoculum, at 60 days after sowing on polyethylene tubes

Substrates			Inoculum		
	B. viridifuturiUFLA 0384	A. spaniusUFRB FA34C2-2	B. cereusUFRB FA72A2-1	With Che	No Che
Chlorophyll a					
SFR	37.23Aa	36.13Ba	32.31Da	35.17Ca	29.84Ea
SF	35.83Ab	33.23Bb	30.45Cb	33.68Bb	28.00Db
SR	32.84Ac	28.43Bc	27.85Bc	32.52Ac	25.29Cc
Pure soil	27.92Ad	24.12Cd	24.22Cd	25.01Bd	22.45Dd
CV (%)	2.38	2.38	2.38	2.38	2.38
Chlorophyll b					
SFR	15.28Aa	10.95Ca	11.04Ca	12.72Ba	12.72Ba
SF	14.90Aa	9.29Db	9.99Cb	10.50Bb	10.50Bb
SR	13.86Ab	9.20Cb	9.28Cc	9.94Bc	9.94Bc
Pure soil	12.78Bc	8.68Dc	13.41Ad	9.23Cd	9.23Cd
CV (%)	4.74	4.74	4.74	4.74	4.74
Total Chlorophyll					
SFR	52.51Aa	47.08CEa	43.35Da	47.89Ba	38.82Ea
SF	50.73Ab	42.52Cb	40.44Db	44.18Bb	36.59Eb
SR	46.70Ac	39.20Cc	37.13Cc	42.46Bc	33.42Ec
Pure soil	40.70Ad	32.80Dd	37.63Bc	34.24Cd	29.45Ed
CV (%)	2.13	2.13	2.13	2.13	2.13
N accumulation (mg plant⁻¹)					
SFR	43.78Aa	17.51Da	21.64Ca	33.32Ba	8.64Ea
SF	24.81Bb	12.99Cb	14.19Bb	14.45Bb	6.10Db
SR	13.84Ac	4.35Cc	4.32CBc	6.62Bc	2.46Dc
Pure soil	6.92Ad	2.42Cd	2.30Cd	3.12Bd	1.64Cd
CV (%)	3.47	3.47	3.47	3.47	3.47
P accumulation (mg plant⁻¹)					
SFR	7.78Aa	3.31Ba	3.69Ba	6.90Ba	0.91Da
SF	6.23Ab	3.16Ba	2.94Ba	3.18Bb	0.76Ea
SR	3.26Ac	1.78Bb	1.18Bb	1.46Bc	0.38Db
Pure soil	1.81Ad	0.51Bb	0.51Bb	0.74Bc	0.27Cb
CV (%)	10.93	10.93	10.93	10.93	10.93
K accumulation (mg plant⁻¹)					
SFR	5.00Aa	2.39Ca	3.02Ba	4.90Aa	1.55Da
SF	2.38Ab	1.71Bb	1.66Bb	1.81Bb	1.18Ba
SR	1.62Ac	0.75Bc	0.54Bc	0.95Bc	0.54Bb
Pure soil	1.06Ad	0.38Bc	0.33Bc	0.56Bc	0.29Bb
CV (%)	19.63	19.63	19.63	19.63	19.63

SFR = soil + organic fertilizer + rock powder; SF = soil + organic fertilizer; SR = soil + rock powder. Means followed by the same uppercase letter in a row belong to the same group according to the Scott–Knott test at the 5% probability level; means followed by the same lowercase letter in a column do not differ significantly according to the Tukey test at the 5% probability level. With Che: with chemical fertilization; No Che: without chemical fertilization and without inoculation

Substrates composed of soil + organic fertilizer + silicate rock powder and soil + organic fertilizer, when combined with bacterial inoculation particularly with strain *B. viridifuturi* UFLA 03–84 enhanced the nutritional status of the seedlings, resulting in significantly higher accumulations of nitrogen (N), phosphorus (P), and potassium (K) (*p* < 0.05) (Tables 5 and 6).

In Experiment 1, seedlings grown in these substrates and inoculated with *B. viridifuturi* UFLA 03–84, *(A) spanius* UFRB FA34C2-2, *(B) cereus* UFRB FA72A2-1, or supplemented with mineral nitrogen exhibited the highest K accumulation (Table 5).

In Experiment 2, seedlings cultivated in soil + organic fertilizer + silicate rock powder, inoculated with *B. viridifuturi* UFLA 03–84, and supplemented with chemical fertilization showed no significant difference in K accumulation when compared to each other (Table 6).

**Table 6:** Averages of the nutritional variables of *Passiflora edulis* Sims seedlings based on the interaction between the cultivation substrate and inoculum, at 90 days after sowing on polyethylene bags

Substrates			Inoculum		
	B. viridifuturiUFLA 0384	A. spaniusUFRB FA34C2-2	B. cereusUFRB FA72A2-1	With Che	No Che
Chlorophyll a					
SFR	45.66Aa	40.69Ca	40.40Ca	44.15Ba	29.90Da
SF	39.73Ab	35.55Bb	34.12Bb	39.90Ab	28.60Db
SR	35.26Ac	30.76Cc	29.61Dc	34.06Bc	27.57Ec
Pure soil	29.99Ad	28.44Bd	26.18Cd	26.03Cd	23.95Dd
CV (%)	2.25	2.25	2.25	2.25	2.25
Chlorophyll b					
SFP	20.31Aa	19.14Ba	16.15Db	17.73Ca	11.08Ea
SF	19.74Ab	13.98Db	18.06Ba	16.43Cb	9.05Eb
SR	12.19Ac	12.21Ac	11.95Ac	11.01Bc	9.08cb
Pure soil	10.99Ad	10.06Ad	10.66Ad	9.93Bd	9.19Cb
CV (%)	3.19	3.19	3.19	3.19	3.19
Total Chlorophyll					
SFR	65.92Aa	59.83Ca	56.15Da	61.86Ba	40.96Ea
SF	59.46Ab	49.53Db	52.23Cb	56.33Bb	37.65Eb
SR	47.45Ac	42.98Cc	41.54Dc	45.08Bc	36.65Ec
Pure soil	40.98Ad	38.50Bd	36.84Cd	35.95Cd	33.14Dd
CV (%)	1.96	1.96	1.96	1.96	1.96
N accumulation (mg plant⁻¹)					
SFR	92.49Aa	85.40Aa	80.79Ba	76.84Ba	21.22Da
SF	84.07Ab	57.11Cb	52.32Cb	63.01Bb	15.19Da
SR	34.78Ac	26.03Bc	20.90Bc	28.95Ac	5.34Cb
Pure soil	20.00Ad	16.18Ad	9.25Bd	15.18Bd	4.36Bb
CV (%)	10.54	10.54	10.54	10.54	10.54
P accumulation (mg plant⁻¹)					
SFR	28.05Aa	19.84Ca	21.17Ca	23.44Ba	4.65Da
SF	23.80Ab	12.97Cb	11.10Cb	15.56Bb	3.33Dab
SR	7.91Ac	5.47Ac	4.16Bc	6.77Ac	1.41Cab
Pure soil	4.98Ac	4.26Ac	2.29Bc	3.99Ac	4.65Db
CV (%)	15.28	15.28	15.28	15.28	15.28
P accumulation (mg plant⁻¹)					
SFR	13.75Aa	9.92Ba	12.29Aa	12.88Aa	3.03Ca
SF	11.09Bb	7.72Bb	6.14Cb	8.62Bb	2.23Dab
SR	4.01Ac	2.99Bc	2.13Cc	4.01Ac	0.83Dbc
Pure soil	2.57Ad	2.32Ac	1.16Bc	2.15Ad	0.56Bc
CV (%)	13.07	13.07	13.07	13.07	13.07

SFR = soil + organic fertilizer + rock powder; SF = soil + organic fertilizer; SR = soil + rock powder. Means followed by the same uppercase letter in a row belong to the same group according to the Scott–Knott test at the 5% probability level; means followed by the same lowercase letter in a column do not differ significantly according to the Tukey test at the 5% probability level. With Che: with chemical fertilization; No Che: without chemical fertilization and without inoculation

Seedlings inoculated with *(A) spanius* UFRB FA34C2-1 and *(B) cereus* UFRB FA72A2-1 exhibited significantly higher nutrient accumulation compared to the treatment without inoculation and chemical fertilization in both experiments (Tables 5 and 6). Lower accumulations of N, P, and K were observed in seedlings grown in pure soil, regardless of inoculation.

In Experiment 1, no significant difference in phosphorus accumulation was observed between the substrates composed of soil + silicate rock powder and pure soil (Table 5). The lowest averages for these variables were recorded in the treatment without inoculation and chemical fertilization.

## Discussion

All bacterial strains demonstrated the ability to convert insoluble minerals, including silicon (Si), potassium (K), and sodium (Na), into soluble forms, with varying degrees of efficiency. *Bradyrhizobium viridifuturi* UFLA 03–84 showed outstanding performance in increasing Si and K availability in the culture medium (Figs. 4 and 5). These results suggest that strain *B. viridifuturi* UFLA 03–84 acts as a silicate-solubilizing bacterium capable of enhancing the benefits of rock powder application. Given that the rock powder used in this study originated from pyroxenite, a mafic–ultramafic silicate rock rich in ferromagnesian minerals such as pyroxenes, its mineralogical composition is particularly relevant.

Additionally, strain *B. viridifuturi* UFLA 03–84 solubilized Si and K in alkaline media with high electrical conductivity (Figs. 3 and 4, and 5). In other studies, this strain was also shown to solubilize Ca_3_(PO_4_)_2_ [36] and tolerate up to 30 g L⁻¹ of NaCl in culture media [39]. Nutrient solubilization by rhizobacteria is a promising strategy for improving nutrient availability to plants [2, 23, 29]. However, further research is necessary to validate the effectiveness of these bacteria in enhancing nutrient availability under controlled conditions over time and in various cultivation and substrate contexts for seedling production using silicate rock powder and other microbial agents.

Several studies have suggested that the decrease in pH during fermentation, resulting from organic acid production and proton release, is a key mechanism for phosphate solubilization [20, 36, 49]. However, strains *B. viridifuturi* UFLA 03–84, *Achromobacter spanius* UFRB FA34C2-2, and *Bacillus cereus* UFRB FA72A2-1 did not reduce the initial pH of 6.8 after 35 days of fermentation (Fig. 2), indicating that acidification was not the mechanism responsible for solubilization at this pH. It is possible that the acids were present in anionic forms, contributing instead to Si chelation [25]. Only strain UFLA 03–84 demonstrated effective solubilization of Si (16 mg L⁻¹) and K (16.5 mg L⁻¹), along with an increase in pH (Figs. 3 and 4, and 5), suggesting the involvement of alternative solubilization mechanisms such as siderophore production, exopolysaccharide (EPS) secretion, or silicase enzyme activity [5, 17, 20, 36, 44]. Therefore, the bacterial capacity to solubilize minerals reinforces the agronomic value of both the inoculant and the rock powder.

An increase in soluble Na⁺ concentration was observed in the nutrient broth (Fig. 4), a result also reported by Setiawati and Mutmainnah [49] when evaluating mineral-solubilizing bacteria on Alexandrov agar medium. During the solubilization of silicate rock powder, protons and organic acids such as ferulic, citric, and coumaric acids are released, acidifying the medium, destabilizing mineral structures [20, 49], and increasing the availability of elements present in the rock (Figs. 4 and 5). Under saline stress conditions, strain *B. viridifuturi* UFLA 03–84 has demonstrated tolerance to a wide range of pH values (4 to 10), temperatures (15 to 37 °C), and NaCl concentrations (up to 0.75%) [16, 39], highlighting its adaptability to diverse soil and climate conditions in Brazil [16, 33, 55, 56].

Inoculation with strain *B. viridifuturi* UFLA 03–84 in combination with soil, organic fertilizer, and rock powder improved all morphophysiological traits and nutrient status of *Passiflora edulis* Sims seedlings. These treatments outperformed the combination of soil, organic fertilizer, rock powder, and chemical fertilizer (Tables 3, 4 and 5, and 6), regardless of substrate volume or cultivation period. The benefits of inoculation may be attributed to the strain’s ability to produce auxins, amylase, and catalase (Table 2), making these compounds available to the plant. These findings indicate that inoculation with this strain, along with organic fertilizer and rock powder, met the nutritional requirements at this developmental stage and may reduce the duration of nursery cultivation.

The observed improvements in morphophysiological traits and nutrient content in *P. edulis* seedlings (Tables 3, 4, 5 and 6) can also be explained by changes in the chemical and physical properties of the substrates, including pH, cation exchange capacity, organic matter content, moisture, and water retention [8, 37]. Even in substrates with a low organic matter-to-soil ratio (e.g., 20:80), fertility tended to increase, reaching high levels of P and K. In general, substrates containing 10% to 30% organic matter provide the best results [8, 37]. In this study, improvements were observed in the 2:1 (soil: organic compost) substrate, regardless of the bacterial strain used.

The benefits of plant growth-promoting bacteria (PGPB) largely depend on the affinity between microorganisms and plant species. In this study, plant growth promotion was closely linked to root colonization, particularly in seedlings inoculated with *B. viridifuturi* UFLA 03–84 followed by *(A) spanius* UFRB FA34C2-2. The increase in root length resulting from inoculation with *(B) viridifuturi* UFLA 03–84 and cultivation in soil enriched with organic fertilizer and rock powder (Tables 3 and 4) likely provided structural support and greater seedling vigor under field conditions, balancing shoot growth and increasing resistance to mechanical stress. These morphological changes may also result from enhanced P availability via microbial solubilization in the substrate [21].

Inoculation with PGPB improved nutrient availability in cultivation substrates, especially those containing organic fertilizer and silicate rock powder, as well as organic fertilizer alone (Tables 5 and 6). This improvement in plant nutritional status resulted from organic matter mineralization [1] and the gradual nutrient release from rock powder, a process accelerated by the presence of solubilizing bacteria [50].

It is also important to consider that the organic fertilizer used in this study may have introduced a diverse community of native microorganisms capable of influencing nutrient solubilization and plant development. Organic fertilizers are known to harbor beneficial microbial consortia, including bacteria and fungi with functional traits such as phosphate and silicate solubilization, nitrogen fixation, and the production of phytohormones and exopolysaccharides [58]. Therefore, the improved morphophysiological and nutritional parameters observed in treatments containing organic fertilizer, either alone or combined with rock powder, may result not only from the nutrient content of the organic material but also from the synergistic activity between the native microbiota and the inoculated strains. This potential interaction could have enhanced mineral solubilization and nutrient uptake by the plants, reinforcing the importance of further studies to distinguish the individual and combined effects of native and inoculated microorganisms under controlled conditions.

The positive effects of *B. viridifuturi* UFLA 03–84 on N, P, and K accumulation (Tables 5 and 6) can be attributed to biological nitrogen fixation [13], nutrient solubilization [2, 20], and extracellular enzyme production (Table 2). Other unquantified microbial processes, such as exopolysaccharide secretion, may also have contributed to these effects [23, 45].

Root architecture influences water and nutrient absorption capacity [6]. Root elongation, often triggered by nutrient deficiency, directs energy toward nutrient acquisition, resulting in longer roots and reduced shoot growth. This pattern was observed in seedlings cultivated on soil + rock powder and pure soil (Tables 3 and 4).

The Dickson Quality Index (DQI) [19], which considers plant robustness and biomass distribution, showed significantly higher values in seedlings inoculated with *B. viridifuturi* UFLA 03–84 and cultivated in soil + organic fertilizer + rock powder. These improvements may be due to enhanced nutrient availability (Figs. 3 and 5; Tables 3 and 5), hormone secretion, and improved water and nutrient uptake, resulting in greater plant growth and biomass accumulation [42, 43]. Combined bacterial inoculation and organomineral fertilization has been reported to reduce nitrogen fertilizer requirements, improve nutrient availability in *Vicia faba* L., and increase soil nitrogen content (Hannon et al., 2020). These findings corroborate the present study, suggesting that nitrogen fertilization can be reduced or replaced in *P. edulis* seedling production using organomineral substrates.

Seedlings grown in soil-only substrates showed lower nutrient uptake, reduced growth and biomass, and lower DQI values compared to those cultivated with organic fertilizer and silicate rock powder, with a 75% improvement (Tables 3 and 4). The Dystrophic Yellow Latosol used in this study is acidic and nutrient-poor, emphasizing the importance of substrate fertilization to ensure seedling quality through adequate nutrition, root development, and microbial activity [32].

Nitrogen, phosphorus, and potassium fertilization improved seedling growth and nutrition at 60 and 90 DAS compared to treatments without mineral N and inoculation (Tables 3, 4, 5 and 6). In other studies, applying 600 mg dm⁻³ of mineral K increased seedling height and total dry biomass (Myake et al., 2016). However, inoculation with *B. viridifuturi* UFLA 03–84 combined with organic fertilization and rock powder may provide comparable gains while reducing chemical fertilizer inputs.

This study clearly demonstrated increased nutrient content in seedlings grown in substrates enriched with rock powder and organic fertilizer. In the context of sustainable agriculture, these results are timely and highlight the potential to improve soil health through alternative substrate formulations that promote the sustainable production of high-quality seedlings.

Bioinputs play a critical role in sustainable development by transforming agriculture through low-impact practices aligned with the Sustainable Development Goals (SDGs). By integrating living organisms, organic fertilization, and biological processes, bioinputs enhance plant nutrition, suppress pests and diseases, and reduce dependence on synthetic inputs, major contributors to soil degradation and water contamination. This approach aligns directly with SDG 2 (Zero Hunger and Sustainable Agriculture), SDG 6 (Clean Water and Sanitation), and SDG 15 (Life on Land), promoting environmental conservation and efficient food production. The application of silicates demonstrates how innovative practices can improve plant nutrition. Silicates are solubilized in soil through root exudates and microbial action, particularly from silicate-solubilizing microorganisms [26, 59], increasing Si availability for plant uptake and enhancing stress resilience [18, 31, 43]. In *P. edulis* cultivation, the combined use of biological inoculants and rock powder supported vigorous plant development and improved sustainability in production systems. This biotechnology thus represents an innovative solution aligned with the SDGs, fostering food security, soil conservation, and sustainable agricultural practices.

## Conclusions

The bacterial strains *Bradyrhizobium viridifuturi* UFLA 03–84, *Achromobacter spanius* UFRB FA34C2-2, and *Bacillus cereus* UFRB FA72A2-1 demonstrated the ability to solubilize nutrients from silicate rock powder, revealing their potential as bioinputs for sustainable agriculture. Among them, *B. viridifuturi* UFLA 03–84 exhibited superior efficiency in solubilizing silicon and potassium and promoted significant improvements in the morphophysiological and nutritional quality of *Passiflora edulis* seedlings. Seedlings cultivated in a substrate composed of soil + organic fertilizer + rock powder (2.08 g dm⁻³) and inoculated with this strain showed higher nutrient accumulation and better growth performance at both 60 and 90 days of cultivation, making this combination a promising biotechnological alternative for passion fruit seedling production.

This study advances the understanding of how microbial inoculation can enhance the agronomic efficiency of rock powder and organic fertilizers, reducing dependence on chemical fertilizers. The results also highlight the need for further investigations to elucidate the synergistic interactions between inoculated strains and native microorganisms introduced through organic fertilizers, as well as to evaluate the persistence, colonization dynamics, and efficiency of these bacteria under field conditions. Such studies will strengthen the application of microbial–mineral–organic integrations in sustainable and low-input agricultural systems.

## Data Availability

All data are provided in the manuscript.
